# Group Behavioural Responses of Atlantic Salmon (*Salmo salar* L.) to Light, Infrasound and Sound Stimuli

**DOI:** 10.1371/journal.pone.0063696

**Published:** 2013-05-17

**Authors:** Samantha Bui, Frode Oppedal, Øyvind J. Korsøen, Damien Sonny, Tim Dempster

**Affiliations:** 1 Sustainable Aquaculture Laboratory – Temperate and Tropical (SALTT), Department of Zoology, University of Melbourne, Victoria, Australia; 2 Institute of Marine Research, Matredal, Norway; 3 ProFish Technology, Jambes (Namur), Belgium; 4 Centre for Research-based Innovation in Aquaculture Technology (CREATE), SINTEF Fisheries and Aquaculture, Trondheim, Norway; Universitat de Barcelona, Spain

## Abstract

Understanding species-specific flight behaviours is essential in developing methods of guiding fish spatially, and requires knowledge on how groups of fish respond to aversive stimuli. By harnessing their natural behaviours, the use of physical manipulation or other potentially harmful procedures can be minimised. We examined the reactions of sea-caged groups of 50 salmon (1331±364 g) to short-term exposure to visual or acoustic stimuli. In light experiments, fish were exposed to one of three intensities of blue LED light (high, medium and low) or no light (control). Sound experiments included exposure to infrasound (12 Hz), a surface disturbance event, the combination of infrasound and surface disturbance, or no stimuli. Groups that experienced light, infrasound, and the combination of infrasound and surface disturbance treatments, elicited a marked change in vertical distribution, where fish dived to the bottom of the sea-cage for the duration of the stimulus. Light treatments, but not sound, also reduced the total echo-signal strength (indicative of swim bladder volume) after exposure to light, compared to pre-stimulus levels. Groups in infrasound and combination treatments showed increased swimming activity during stimulus application, with swimming speeds tripled compared to that of controls. In all light and sound treatments, fish returned to their pre-stimulus swimming depths and speeds once exposure had ceased. This work establishes consistent, short-term avoidance responses to these stimuli, and provides a basis for methods to guide fish for aquaculture applications, or create avoidance barriers for conservation purposes. In doing so, we can achieve the manipulation of group position with minimal welfare impacts, to create more sustainable practices.

## Introduction

Mapping the flight and avoidance responses of animals to aversive stimuli has led to conceptual advances in behavioural sciences, including understanding predator-response behaviours [1] and the evolutionary development of variable flight behaviour [2]. Theoretical studies investigate the ecological implications of flight behaviour performance [3] and predator-prey interactions [4], and determine the types of cues that induce such behaviours [5]. Applied studies of flight behaviour pursue a means of influencing the movement and position of the fish by harnessing the natural behaviours of groups of fish. Various external stimuli have been used to manipulate fish behaviour, such as stroboscopic light [6], [7], infrasound [8], [9], bubble curtains [10], and other physical cues ([11], see [12] for review).

How information from the environment is received and processed by an individual depends on their sensory capabilities, and hence, responses to extrinsic signals are species-specific [13]. Insight into the anatomical, physiological, and neural sensitivities of a species assists in understanding their perception of signals, and thus the mechanisms that elicit responses [5]. We can use this knowledge to construct methods to guide fish behaviour that can be useful for conservation management or finfish aquaculture. For instance, it would benefit the development of fish deterrent systems to minimise mortality or entrainment in hazardous areas [14], or for novel methods of influencing the distribution of fish in aquaculture (e.g. [15]), where manipulating the vertical position of thousands of individuals without harm remains a challenge.

The environment is saturated with visual indicators, and teleost fish are highly adapted to detect changes in the visual environment [16]. The intensity, spectral composition, and polarisation of light are factors that influence salmonid vision [17]. Salmonids detect polarised light and are sensitive to light of varying spectral composition including ultraviolet, blue, green, yellow, and red (range of 346 nm to 690 nm; [18]). They have a strong behavioural response to acute changes in the light environment; four species of salmonids dived immediately to the bottom of tanks and swam with elevated activity after a transition from light-to-dark or dark-to-light environments [19]. Abrupt exposure to artificial light also elicits strong avoidance responses across taxa, including rainbow smelt (*Osmerus mordax*; [20]), zebrafish (*Danio rerio*; [7]), yellow perch (*Perca flavescens*), largemouth bass (*Micropterus salmoides*), coho salmon (*Oncorhynchus kisutch*) and chinook salmon (*Oncorhynchus tshawytscha*; [6], [21]).

Sound has been explored as a potential behavioural modifier, and has been suggested as a better candidate than light [11]. Salmonids do not have special adaptations for hearing [22] however Atlantic salmon (*Salmo salar* L.) are sensitive to acoustic particle motion, particularly at frequencies below 200 Hz [23] and even more so for sounds well below 50 Hz [24], [25]. Salmon avoid infrasound frequencies in freshwater environments (5–10 Hz; [8], [26], [27]); the use of infrasound to elicit avoidance responses was trialled with success in juvenile chinook salmon and rainbow trout (*Oncorhyncus mykiss*; [28]), cyprinids [14], and European eels (*Anguilla anguilla*; [29]). The mechanisms driving these responses are unclear, however the use of low frequency signals in the sound environment is common in communication [30], [31], and may be analogous to the frequency produced by their predators [8], [32]. Fish may also be acutely sensitive to particle displacement generated by breaking the surface of the water [11], [33] due to anticipation of predator activity, such as from birds or seals [32], [34].

A species will often have a pronounced response upon exposure to a novel, high intensity, or aversive stimulus; this response will always be within its normal behavioural repertoire, and a common reaction to a potentially harmful signal is to escape and gain distance away from the source (e.g. [35]). In fish, the flight response is often fleeing to deeper waters [1], [18]. Flight behaviours are characterised by fast-start swimming: a high-energy burst and rapid acceleration in swimming speed [36], [37], usually in the direction away from the disturbance [38]. The duration of stress responses are a trade-off between the potential risk represented by the signal and the cost of avoidance [4], and thus how long a stimuli elicits an effect is indicative of the magnitude of stress induced.

Fundamental behavioural experiments are commonly conducted in tanks and aquaria in the laboratory, on individuals and small groups of fish. However, their relevance requires investigation with large groups of fish in field settings, with carefully monitored environmental conditions. Here, we investigated the avoidance behaviours of groups of Atlantic salmon held in a marine environment, by characterising immediate behavioural responses and short-term effects to aversive stimuli. Fish were acutely exposed to light of different intensities, to infrasound, and to surface disturbance. The depth at which the group were swimming in the water column was monitored over time, as well as level of acoustic backscatter from the group. Behavioural responses measured included surface activity and swimming speed.

## Materials and Methods

### Ethics Statement

The work was conducted in accordance with the laws and regulations of the Norwegian Regulation on Animal Experimentation 1996. The protocol was approved by the Norwegian Animal Research Authority (Ethics permit number: 3619; local responsible: Tom Hansen, IMR).

### Location and Experimental Set-up

The experiments were conducted at the Cage Environment Laboratory at the Institute of Marine Research, in Masfjorden, western Norway (60° N). Light experiments ran from August 7 to August 27, while sound experiments ran from August 22 to September 3, 2011 (hereafter referred to as the experimental periods). Two experimental cages (5 m×5 m×5 m;≈125 m^3^ volume) were used for light experiments with a tarpaulin suspended underwater between experimental cages to avoid light contamination. A single cage of the same dimensions was used in sound trials.

#### Light experiments

**Figure 1:**
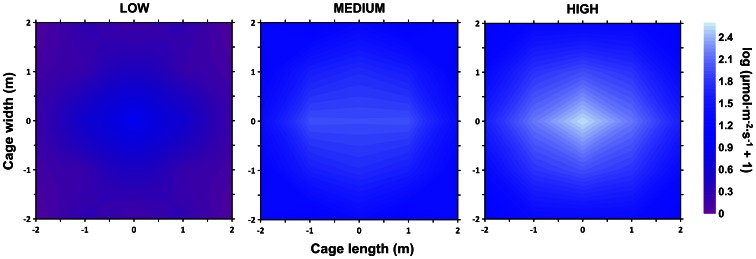
Distribution of light emitted from a 400 W blue LED lamp for three intensities of light (low, medium and high). Measurements of intensity (µmol·m^−2^·s^−1^) were determined using an underwater spherical quantum sensor at 2 m depth in the sea-cage, and were log(X+1) transformed. Coordinates (0, 0) indicate the centre of the cage.

A submersible light-emitting diode (LED) lamp (prototype provided by AKVA Group, Bryne, Norway) was suspended in the centre of the cage at a depth of 1.5 m, emitting a blue light (peak at 460 nm; colour temperature of 20,000 K) with a power rating of 400 W. Lamps currently used in farmed settings utilise metal halide bulbs (150–1000 W) which emit light that gradually increases in intensity when turned on, however LED lamps can provide immediate, brilliant illumination. Light intensities were measured using an underwater spherical quantum sensor LI-193SA connected to a LI-1400 data logger (Li-Cor, Lincoln, NE, USA). The three intensities used as treatments were defined as low (0.8 µmol·m^−2^·s^−1^), medium (26.8 µmol·m^−2^·s^−1^) and high (35.4 µmol·m^−2^·s^−1^) intensity (Fig. 1). The registered photosynthetic photon flux fluence rates for each intensity were measured 0.5 m from the lamp. The lamp remained in the water throughout the experimental period, including acclimatisation periods, to minimise confounding effects. The sampling period was run once there was complete darkness (when the contrast between the stimulus and ambient illumination would be the greatest), and thus began between the hours of 0∶00 and 0∶30.

For a more detailed light environment description, intensities were also measured during the night at 1 m intervals on the horizontal and vertical planes (horizontal plane at 2 m depth only shown in Fig. 1). The intensities and the wavelength of the light emitted in the treatments are within the range of visual sensitivities for Atlantic salmon [17].

#### Sound experiments

The sound trials used an industrial infrasound source (ProFish Technology, Belgium; described in [14]), which was designed as an integrated system that emits a low frequency sound to deter fish from hazardous areas (such as cooling water intakes at nuclear power stations). The infrasound device was suspended in the centre of the cage and submerged at 2 m depth, remaining there for the entire experimental period. The machine was set to a frequency of 12.5 Hz and calibrated before each trial. The sound source was controlled from a separate module located out of the water, 15 m away from the cage, ensuring that the treatment would begin with no visual indicator to the fish. To imitate a surface disturbance event, we used a disc (Ø = 30 cm) suspended with ropes 1 m above the surface of the water, near the centre of the cage. During this treatment, the disc was dropped flat against the water, creating a ‘slapping’ effect. It was then pulled up, and this procedure was repeated every 10 s. Sampling periods began between 10∶00 and 15∶00.

### Experimental Design

In both light and sound experiments, all treatments were replicated 3 times. Treatment replicates were interspersed across the 2 experimental cages in the light experiment, and throughout the experimental periods in light and sound experiments, to avoid possible confounding due to environmental variability.

#### Light experiment

To test the responses of the fish to acute exposures of intense light, we conducted experiments where groups of fish were subjected to different intensities of light. The design included a control (LC), where the lamp was present but no light was emitted, and exposure to either high (LH), medium (LM) or low (LL) intensity light. We measured the behaviours of salmon in four experimental periods; ‘Before’ (B) the 10 minutes before the treatment was applied; ‘During’ (D) when the light cues were applied for 10 min; and two ‘After’ periods, 0–10 min and 10–20 min after the cues had ceased (A1 and A2, respectively). In D, the LED light was turned on and maintained at the relevant intensity for 10 min.

#### Sound experiment

To investigate how fish respond to a variety of sound components, the sound trials included a control (SC); the use of the infrasound source (SI); the effect of surface disturbance using the disc (SS); and both the infrasound and surface disturbance (SB) occurring simultaneously. As in the light experiment, we measured the behaviours of salmon in four experimental 10 min periods (B, D, A1, and A2). In D, infrasound was applied for 1 min, followed by 1 min when it was off, and repeated in this manner throughout the 10 min period. Similarly, surface disturbance treatments were conducted with 1 min of the disc being dropped continuously (every 10 s), followed by 1 min with no action, for 10 min.

### Experimental Fish

Fish were sourced from a full-scale production cage (∼ 2000 m^3^), located 5–15 m away from the experimental cages. Before each replicate, a group of fish was crowded in the production cage using a 5 m×5 m×5 m cast net to capture a large sample group and bring them to the surface, where 50–53 fish were then randomly caught using a dip net and transferred to the experimental cage. The fish were allowed to recover for a minimum of 24 h, and were not fed during their time in the experimental cage. Upon completion of the treatment, 10 fish were randomly netted out, anaesthetised with Benzoak VET (dose: 0.2 ml L^−1^ of seawater), then measured for total length and weight.

A total of 1219 Atlantic salmon (*Salmo salar* L.) were used in the two experiments (50–53 fish per replicate×3 replicates×4 treatments×2 experiments). The fish weighed on average 1232±359 g (range: 110 to 2360 g) and 1431±372 g (range: 680 to 2185 g), with a total length of 51.2±4.2 cm (range: 36 to 61 cm) and 53.0±3.9 cm (range: 43 to 61 cm), for the light and sound experiments, respectively. There was no difference between mean weights or lengths among treatments, in both light (one-way ANOVAs; *F*_[3,115]_ = 2.05 and 1.70; *P* = 0.11 and 0.17, respectively) and sound experiments (one-way ANOVAs; *F*_[3,115]_ = 0.02 and 0.27; *P* = 1.0 and 0.86, respectively).

### Group Swimming Depths and Acoustic Backscatter

The swimming depth of the group was continuously recorded using a PC–based echo integration system (Lindem Data Acquisition, Oslo, Norway; described by [39]) connected to transducers positioned below the cage, approximately 7 m deep, facing upwards with a 42° acoustic beam. This gave measures of echo intensity, which is directly related to fish density, at depth intervals of 0.5 m. Outputs of echo strength were given as the mean value of echo intensity per minute, and each cell (in depth and time) was calculated as a proportion of the total acoustic backscatter (sum of echo intensities) received at that time point, across all depths.

An increase in activity of the fish causes changes in tilt angle and distance from the echosounder, thereby decreasing the horizontal projection and consequently total acoustic backscatter. Thus whilst the stimulus is being applied, acoustic backscatter will be highly variable if fish exhibit greater swimming activity. This is expected also of the following period, and so acoustic backscatter values from A2 with more recovered fish are of most interest. Furthermore, the structure and volume of the swim bladder contributes a large proportion of the acoustic backscattering [40], and hence changes in total acoustic backscatter values from A2 will describe any change in the swim bladder volume from its original form in B. The percentage change in total acoustic backscatter was calculated by comparing levels in A2 with initial levels before the treatment began.

### Surface Activity

When the light was turned off at the beginning of period A1, the number of splashes heard was counted in the first minute, and then averaged across two observers. Splashes represented surface activities exhibited in the group, indicating the magnitude of an aversion response to the abrupt change in light.

### Swimming Speeds and Behaviours

Swimming speeds and behaviours were monitored for all four periods of the sound experiment, via an underwater camera (360° pan/tilt Orbit Subsea camera, Norway, www.orbitgmt.com) submerged to the depth of the group. The camera was controlled by winches and recorded video clips throughout the sampling period. Instantaneous swimming speeds were calculated from the video recordings, in body lengths per second (BL·s^−1^) by measuring the time taken for the snout and tail of a fish to pass a vertical reference line in the cage [41]. Each experimental period was divided into three parts, and 10 fish for each third were haphazardly chosen and used for analysis, totalling 30 measurements per period and 120 in the replicate.

### Environmental Variables

Temperature was recorded from 0 to 9 m depth using an online probe (YSI model 30–50 ft, YSI, OH, USA) and a Secchi disc (∅ = 30 cm) was used to quantify water turbidity during the light experimental period only. Recordings were taken every day throughout the experimental periods, at a standard time of day and reference point near the experimental cages. Dissolved oxygen was monitored via the camera positioned at the group’s swimming depth.

### Statistical Analyses

Differences among treatments in temperature, visibility, number of jumps in the light experiment, and total acoustic backscatter values (log-transformed to reduce variances and correct a skewed distribution; [42]) at A2 compared to its starting level at B, were tested for with a one-way analysis of variance (ANOVA). Significant results from ANOVAs were further analysed using Student-Newman-Keuls (SNK) tests to determine differences in group means. Temperature was averaged across depth bands (0–2 m, 3–5 m, and 6–8 m) for analysis. Total acoustic backscatter values were low-pass filtered, where zero values and those outside of the mean ±2 standard deviations were removed, in order to reflect possible total acoustic backscatter values realistically.

The depth at which the maximum echo intensity occurred (depth_max_) at a time point was used, and the average of all depth_max_ points in the period was calculated. These values were used for analyses of vertical distributions across the four experimental periods. Repeated-measures ANOVAs were used to compare differences in depth_max_ and instantaneous swimming speeds, with period as the repeated measure. Significant results from these were further analysed for within-subject factors using pairwise comparisons, applying a Bonferroni adjustment when p-values were small [42]. Planned comparisons (one-way ANOVAs) were conducted comparing the difference among treatments for depth_max_ and swimming speed data, only within the period when stimuli were applied. SNK tests were conducted if significant results arose.

All analyses were only conducted after parametric test assumptions (normality and homogeneity of variances) were evaluated using residual plots, and statistical significance was determined at α = 0.05.

## Results

### Light Experiment

#### Group swimming depths and acoustic backscatter

Upon exposure to the LM and LH treatments, salmon began swimming fast and erratically in multiple directions, with some individuals making contact with the side of the cage and other individuals. However, when exposed to the LL treatment, this behaviour did not occur – the fish did not show a marked increase in swimming behaviour but instead descended slowly away from the light source.

**Figure 2:**
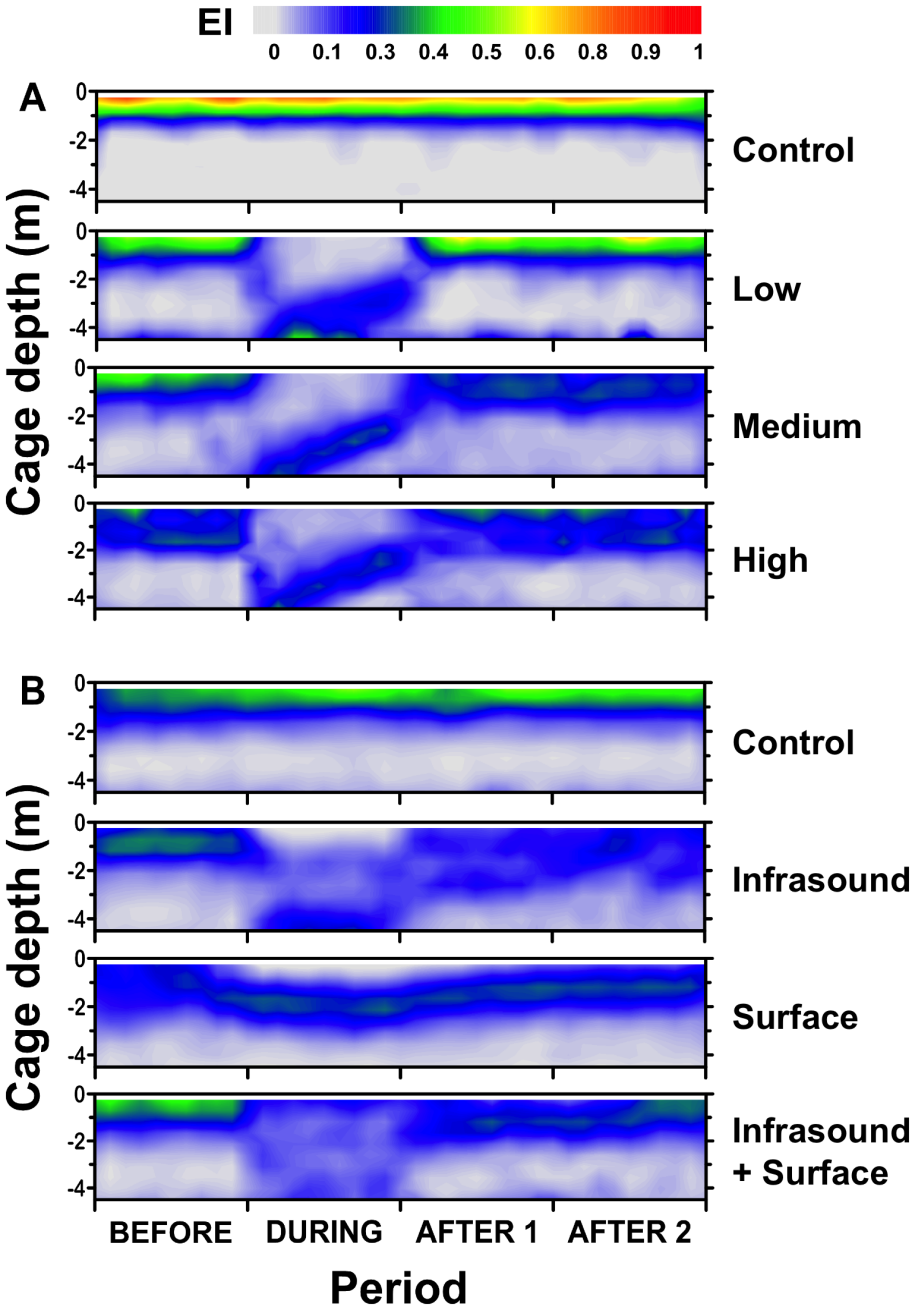
Observed fish densities of Atlantic salmon (*Salmo salar*) in the sea-cage over the experimental period. Echo intensity (EI) was received through an echosounder. Shown are the average for each treatment (*n* = 3) for (A) light and (B) sound trials. The fish are exposed to the stimulus at the beginning of the During period.

In all three treatments, fish dived to the bottom of the sea-cage when the light was turned on. Results from the repeated measures ANOVA revealed that treatments affected swimming depths over the treatment periods (Table 1), and post-hoc pairwise comparisons show that the deeper position of the group in D was different from the other periods (Fig. 2a). Planned comparisons in this period revealed a difference between treatment means at this time (*F* [_3]_, [8] = 96.95, *p*<0.001), with post-hoc SNK tests confirming that the swimming depths during the three light intensities were deeper than Control groups (Fig. 2a). When the light was turned off, the group returned to their surface position in all light treatments (Fig. 2a).

**Table 1:** Summary of repeated measures analysis of variance for the vertical activity of Atlantic salmon (*Salmo salar*), as measured by depth of maximum echo intensity, exposed to light treatments for the 10 min periods before, during, and two periods directly after the stimulus.

Source of variation		SS	*df*	MS	*F*	*p*
Betweensubjects	Treatment	10.59	3	3.53	12.39	0.002
	Residual	2.28	8	0.26		
Withinsubjects	Period	21.15	3	7.05	310.92	**<0.001**
	Period×Treatment	7.37	9	0.82	36.12	**<0.001**
	Residual	0.55	24	0.02		

Bold face values are significant at *p*<0.05.

**Figure 3:**
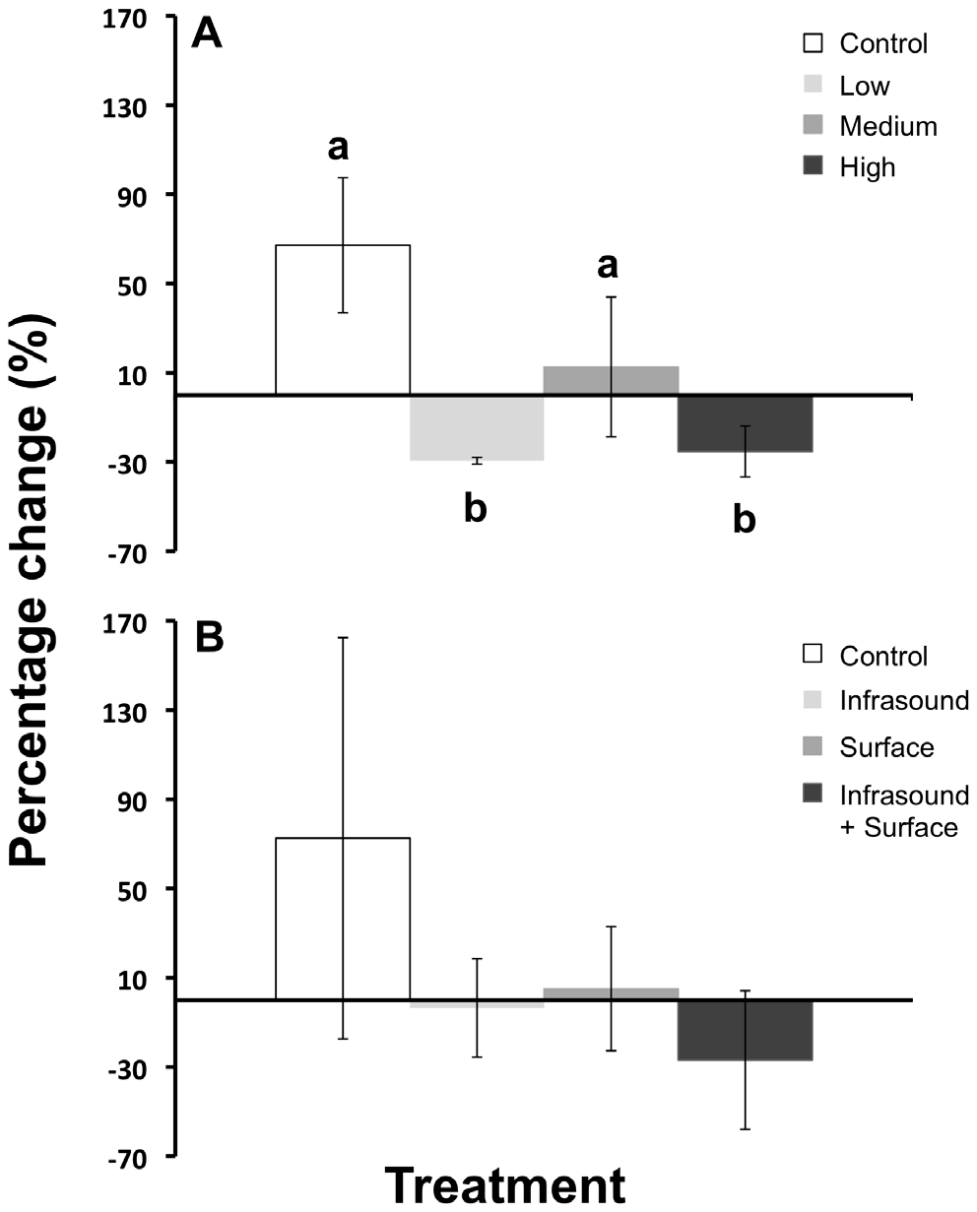
Mean percentage change (± SE, *n* = 3) of total signal strength from the period 10–20 min after the treatment had ceased. The total signal strength from the pre-stimulus period was used as a baseline. Shown are the changes for each treatment in the (A) light and (B) sound trials. Letters (a,b) indicate differences among treatments at *p*<0.05 (as determined by SNK tests).

From observations of the vertical distribution (Fig. 2) and swimming behaviour of the group, the fish have returned to their pre-stimulus state by A2, thus standardising the comparisons of acoustic backscatter between B and A2. There was a decline in average total acoustic backscatter levels for LL (29%) and LH (25%), whereas LC and LM groups increased (67 and 13%, respectively) in acoustic backscatter compared to levels before the stimulus began (*F* [_3]_, [8] = 4.58, *p* = 0.038; Fig. 3a). This was verified as SNK tests separated the change in total acoustic backscatter in Low and High treatment groups from Control and Medium groups.

#### Surface activity

**Figure 4:**
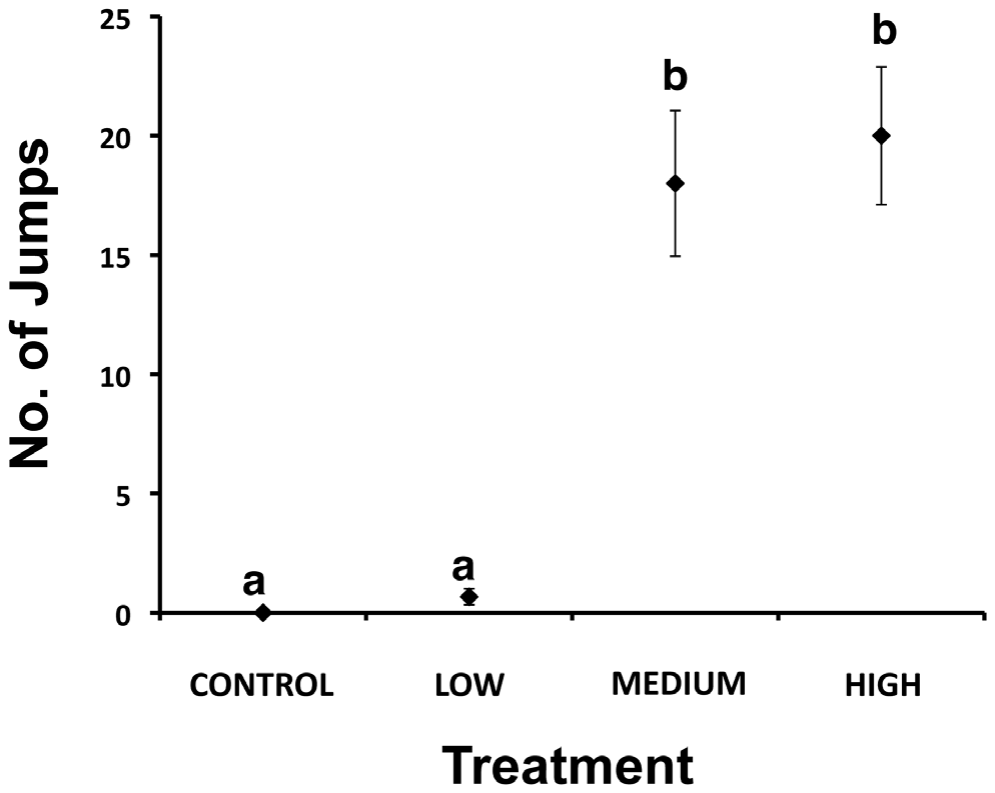
Mean number (± SE, *n* = 3) of jumps observed in Atlantic salmon (*Salmo salar*) following the light treatments. Letters (a,b) indicate differences among treatments at *p*<0.05 (as determined by SNK tests).

Groups exposed to the LH and LM intensities of light jumped on average 18 times more than LL and LC treatments (*F* [_3]_, [7] = 26.30, *p*<0.001; Fig. 4), with SNK tests confirming these discrete groups. Surface behaviours were erratic at the two higher intensities, however activities ceased soon after the 1 min observation period.

### Sound Experiment

#### Group swimming depths and acoustic backscatter

When the infrasound stimulus was applied, salmon swam erratically at their original depth then dived as a group to the bottom of the cage, whereas surface treatment groups responded to the disturbance by actively avoiding the surface and slowly descended to depths of 1.5–2 m (Fig. 2b). When both stimuli were applied together, diving responses were consistent for the first two replicates, but not the last which showed no response (see Figure S1). Nevertheless, when depth_max_ is averaged across replicates, this treatment elicited greater vertical activity in the fish when exposed to the stimuli (Fig. 2b). Analyses showed that treatment influenced swimming depth over the treatment periods (Table 2) and post-hoc tests confirmed that for all treatments, after the disturbance had ceased the group returned to the pre-stimulus swimming depth in A1 and A2 (Fig. 2b). However, swimming depths between treatments, within the D period, were not different from each other (*p* = 0.086). Even so, differences were evident as control groups swam on average at 0.9 m depth, whereas the other groups swam between 2–3 m deep (Fig. 2b).

**Table 2:** Summary of repeated measures analysis of variance for the vertical activity of Atlantic salmon (*Salmo salar*), as measured by depth of maximum echo intensity, exposed to sound treatments for the 10 min periods before, during, and two periods directly after the stimulus.

Source of variation		SS	*df*	MS	*F*	*p*
Betweensubjects	Treatment	4.62	3	1.54	2.17	0.170
	Residual	5.69	8	0.71		
Withinsubjects	Period	6.95	3	2.32	13.68	**<0.001**
	Period×Treatment	4.06	9	0.45	2.663	**0.027**
	Residual	4.06	24	0.17		

Bold face values are significant at *p*<0.05.

The percentage change of total acoustic backscatter in sound experiments was negligible for all treatments, and although there was a small decline in SI and SB groups, the group means were not different among treatments (*p* = 0.675; Fig. 3b). The higher variability may indicate greater vertical spread of individuals within the group (Fig. 2b).

#### Swimming speeds and behaviours

**Figure 5:**
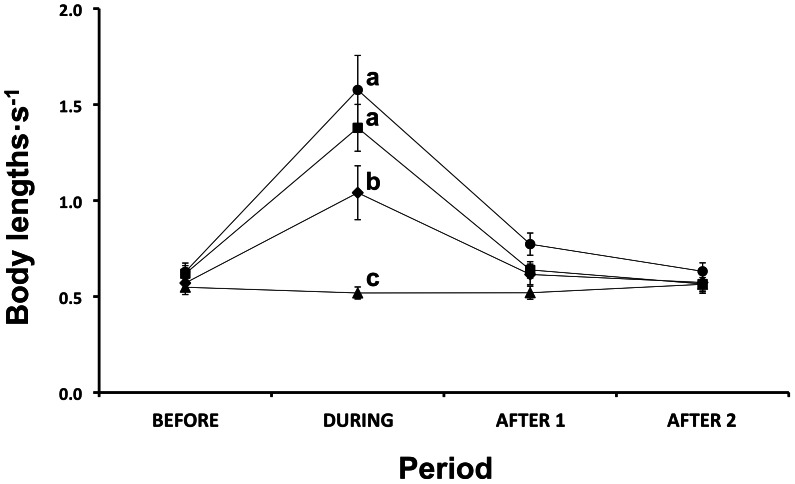
Change in instantaneous swimming speeds of Atlantic salmon (*Salmo salar*) during the experimental sample periods for each sound treatment. Each point represents the mean (± SE) swimming speed, in body lengths per second (BL·s^−1^), of 3 replicates, with the instantaneous swimming speed measured for 30 fish per replicate. Treatments are represented by: ▴, control; •, infrasound; ⧫, surface disturbance; ▪, infrasound and surface disturbance combined. Letters (a, b, c) indicate differences between treatment groups in the period when exposed to stimuli (During), at *p*<0.05 (as determined by a one-way analysis of variance and post-hoc SNK tests).

Infrasound, alone and when combined with surface disturbance, elicited a marked increase in swimming activity in groups of salmon. Treatments modified swimming speeds over the treatment period (Table 3), with swimming speed during the application of stimuli being different from the other three periods when stimuli was not present, confirming that in all treatments the fish had returned to their original swimming speed in A1 and A2 (Fig. 5). Before the treatment period, salmon swam at approximately 0.5–0.6 BL·sec^−1^ in all treatment groups. The application of SI and SB elicited swimming speeds three times greater than that of control groups (Fig. 5), and SS also significantly doubled swimming speeds in this period. During application of the stimulus, the one-way ANOVA test showed differences in swimming speeds (*F* [_3]_, [8] = 9.55, *p* = 0.005). Subsequent SNK tests separated SI, SB and SS groups from SC, and SS from SC (Fig. 5), with the former groups exhibiting faster swimming speeds.

**Table 3:** Summary of repeated measures analysis of variance for the instantaneous swimming speeds (*n* = 90 per treatment) of Atlantic salmon (*Salmo salar*) exposed to sound stimuli, for the 10 min periods before, during, and subsequent two periods after the stimulus was applied.

Source of variation		SS	*df*	MS	*F*	*p*
Betweensubjects	Treatment	0.87	3	0.29	7.48	**0.010**
	Residual	0.31	8	0.04		
Withinsubjects	Period	2.50	3	0.83	45.62	**<0.001**
	Period×Treatment	1.18	9	0.13	7.18	**<0.001**
	Residual	0.44	24	0.02		

Bold face values are significant at *p*<0.05.

### Environmental Variables

Temperature between 0 and 9 m depth did not confound either experiment as it did not differ throughout the experimental period for both the light (*p*>0.05) and sound (*p*>0.05) trials. Temperatures were very similar throughout the depths, with averages of 13.1–14.2°C in light experiments and 14.1–15.5°C in sound experiments. Visibility during the light trial was also consistent over the treatments (*p*>0.05), averaging at 9 m over the trial period (range: 6–12 m). Dissolved oxygen was continuously above 89% for the duration of both experimental periods.

## Discussion

### Light Experiments

The application of light and sound stimuli can influence the vertical position of fish in the ocean. The use of light as a stimulus consistently resulted in a diving response in the fish, where they avoided the light source and swam at a lower depth than their preferred position in the cage. Blue/green light has the highest penetration energy through sea water, and the eyes of salmon can detect light at a minimum intensity of 0.037 µmol·m^−2^·s^−1^ over a wide spectral range [17]; this means that the brilliance of light emitted in the LH power output was in the order of 100 times greater than the lower limit of the salmon eye sensitivity. Thus, the high intensities these fish were exposed to in LM and LH treatments may have been temporarily blinding, as observed through their erratic swimming behaviour and collisions with the net cage. Further, jumping behaviours following the higher intensity light treatments indicated greater aversion to the experience. This was not observed upon exposure to the low intensity light, which suggests the brilliance at the lowest power output was not detrimental to the eye. Nevertheless, the transition from dark to light elicited a pronounced reaction in the fish where the group quickly dived to the bottom of the cage. Our results provide field-based evidence that support previous tank-based experiments, producing dramatic changes in activity and vertical distribution of salmon when lights were turned on [18], [43] and immediate flight away to darker areas upon exposure to bright light [44], [45].

From the aversive responses observed, we would discourage the use of such abrupt changes to the visual environment using high light intensities due to welfare concerns. However, low intensities elicit relatively mild responses and therefore could prove useful in applications requiring short-term behavioural manipulations. With short application of low light stimuli, the behaviour of salmon returns to the pre-stimulus state within 20 min after exposure, indicating the short-term impact of the treatment. Further work is required to investigate the long-term effects of this experience in terms of growth, appetite, body condition, and other welfare parameters.

### Sound Experiments

Infrasound had a similar effect to light, however surface disturbance and combination treatments did not. The behavioural responses were consistent in all trials except for variation in one replicate of the combined treatment (Fig. S1), possibly due to a fault in the infrasound device more so than variable behaviour. Similar to the light experiment, our results reinforce previous tank-based and freshwater experiments on the aversive effect of low frequency sounds to fish, where swimming depth and speeds are influenced by exposure to the sound (e.g. [33]). Knudsen and colleagues [26] determined that infrasound levels (5–10 Hz) were most effective in causing avoidance reactions in Atlantic salmon individuals in a freshwater pool. Previous investigations into the use of infrasound established that fish would produce sudden horizontal flight away from the sound source in rivers [27] and in tanks [28], however fish in aquaculture are restricted in the horizontal plane by the sea-cage and would therefore have to escape downwards, as we have observed. Acoustic cues that could represent threats elicit consistent escape responses in individuals [9], [43], [46] and schools ([37], [47] for summary see [38]). This work represents a positive outcome in that similar to wild cod exposed to acoustic stimuli [48]; the application of infrasound and a surface disturbance event had a short-term effect on salmon behaviours, with fish returning to pre-stimulus states shortly after the cues had ceased. However its effect on flight behaviour may lessen over time as for any stimulus, repeated or extensive exposure can lead to habituation (e.g. [45], [49]), particularly with repeated exposure to infrasound without a visual cue. As such, this approach will be most effective with punctuated, infrequent use.

Disturbance on the surface waters did not produce the flight responses and elevated swimming speeds seen in the other light and sound treatments, only avoidance of the surface. Salmon in aquaculture are constantly exposed to anthropogenic disturbance when farmers conduct maintenance procedures. Therefore, they may be initially frightened by husbandry activities above water (e.g. adjusting the bird net, observing feeding), but become quickly accustomed to it as there is no negative sensations associated with the activity [49]. Flight responses are costly to elicit in terms of energy consumption [4], thus individuals that can distinguish sound components associated with real danger, and reduce responses to false risks, have increased benefits in growth and fitness. If farmed fish are constantly exposed to husbandry events interpreted as predation risk, the welfare of the individual will decline along with appetite and growth [34].

### Practical Implications

The knowledge established from these experiments can be used to develop techniques for fish guidance by eliciting a predictable, natural response through exposure to light or infrasound. Few studies have been conducted on the responses of large groups of fish to infrasound in marine environments, and our results are largely analogous to those that have been done in small tank-based and freshwater experiments. Our results support the assertion that these stimuli can be used in both ocean and freshwater environments to deter fish from infrastructures that represent potential mortalities [12]. Creating behavioural barriers can increase survival of fish populations near hazardous areas, such as turbine inlets for cooling water intakes [14]. Infrasound is an attractive solution in that it is not detectable by humans or fish with restricted sensitivities to sound, reducing its impacts on non-target organisms [11].

In aquaculture or other closed settings, the ability to influence the position of the fish without mechanical manipulation could improve the welfare of farmed fish during farming procedures. One example stems from the salmon aquaculture industry. With the predictable response of salmon to light, new methods can be developed and utilised for numerous farming activities that require the manipulation of the school’s position in the cage. Salmon have a swim bladder that is connected and regulated via the oesophagus [50], requiring them to ‘swallow’ air at the surface to replenish air in the swim bladder, with a behaviour described as jumping or rolling at the surface [15], [51], [52]. A flight response or fast-start swimming may induce the release of air from the swim bladder to facilitate escape or deflect predators [53], [54]. Our findings from the light experiments provide some support for this theory, as exposure to light as an aversive stimulus caused flight into deeper waters. Similarly, the decline in acoustic backscatter for all light intensities, but not in sound experiments, further suggest that there is loss of air from the swim bladder, which reduces the volume for detection by the echosounder [54], [55]. The effect of light stimuli on the change in swim bladder volume has not been investigated previously, and opens new avenues for research on the impact of flight responses on buoyancy in fishes. For instance, this provides a foundation for developing new techniques to treat sea lice in salmon aquaculture, by creating a motivation to break the surface more frequently in order to re-fill the swim bladder and combining this with a layer of floating chemical therapeutant [15]. The application of light stimuli could increase the frequency and intensity of re-filling behaviours, ensuring efficient removal of sea lice.

### Conclusion

Fish in sea-cages are rarely in the same depths at the same time, due to spatial preferences determined by environmental conditions [56] and stocking density [57], however our findings could change this premise. Viable applications of this technique will require developing our understanding on how factors such as age, physical condition, motivation, group size and environmental conditions will affect the behaviour of fishes. Nonetheless, the information gained from this work augments our current understanding of the flight responses of groups of fish to short-term aversive stimuli in a marine environment, and provides a foundation for the use of light and of infrasound to guide fish distributions. This is valuable knowledge for the development of fish guidance methods, and could be further adapted for a wider range of applications in aquaculture or conservation management.

## Supporting Information

Figure S1 **Observed fish densities of Atlantic salmon (** ***Salmo salar*** **) in the sea-cage over the experimental period.** Echo intensity (EI) was received through an echosounder. Shown are the individual replicates from the combination treatment in sound trials, exhibiting the variation between replicates 1 and 2, and replicate 3.
(TIF)
