# Supplementary figures and images for: Group Behavioural Responses of Atlantic Salmon (Salmo salar L.) to Light, Infrasound and Sound Stimuli
Source: PLoS One. 2013 May 17;8(5):e63696. doi: 10.1371/journal.pone.0063696 (PMC3656933; doi:10.1371/journal.pone.0063696)

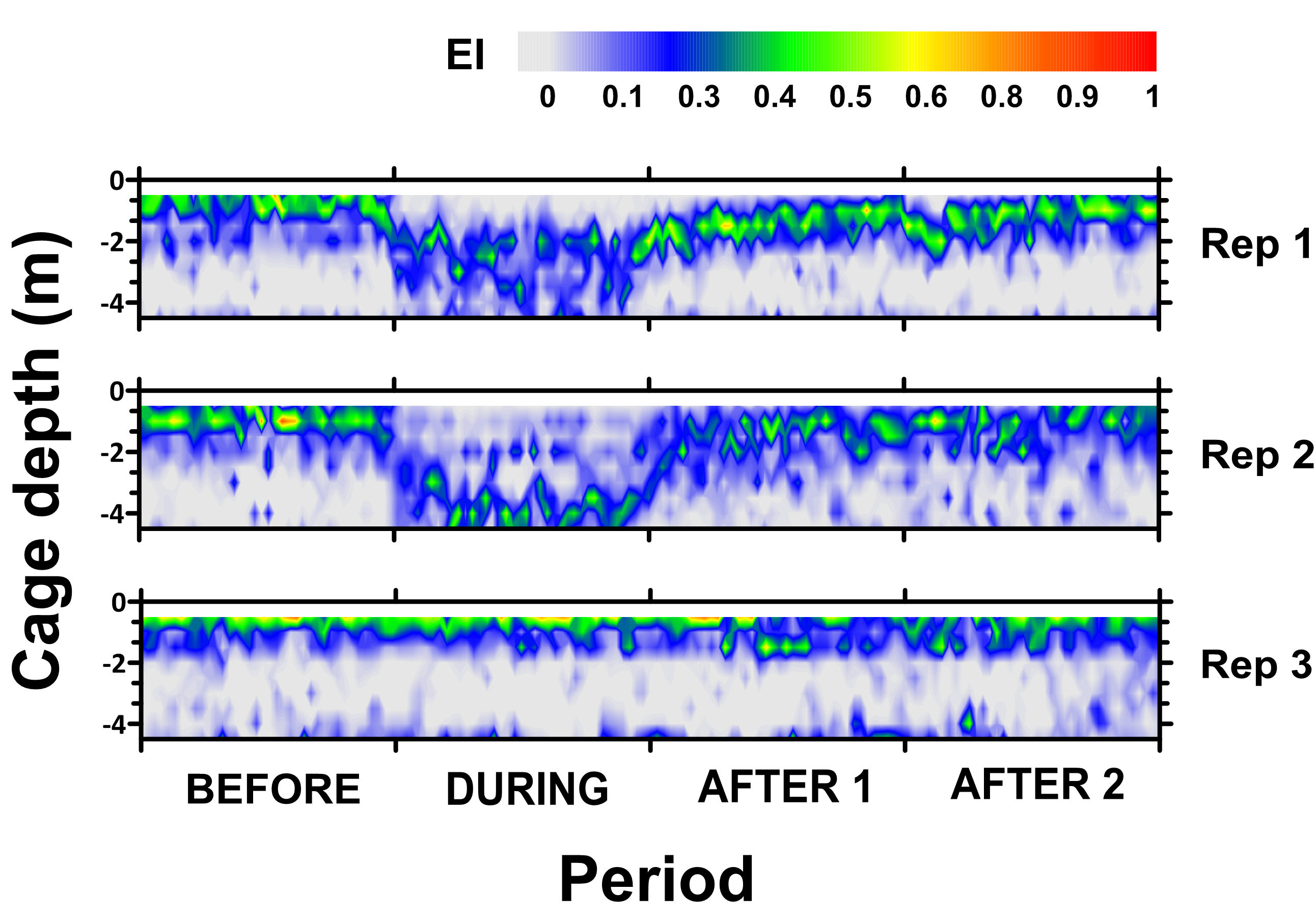

Supplement: Figure S1 — Observed fish densities of Atlantic salmon ( Salmo salar ) in the sea-cage over the experimental period. Echo intensity (EI) was received through an echosounder. Shown are the individual replicates from the combination treatment in sound trials, exhibiting the variation between replicates 1 and 2, and replicate 3. (TIF) [file pone.0063696.s001.tif]
